# Rescue oxygenation success by cannula or scalpel-bougie emergency front-of-neck access in an anaesthetised porcine model

**DOI:** 10.1371/journal.pone.0232510

**Published:** 2020-05-04

**Authors:** Nejc Umek, Iljaz Hodzovic, Marija Damjanovska, Erika Cvetko, Jurij Zel, Alenka Seliskar, Tatjana Stopar Pintaric

**Affiliations:** 1 Institute of Anatomy, Faculty of Medicine, University of Ljubljana, Ljubljana, Slovenia; 2 Department of Anaesthetics, Intensive Care and Pain Medicine, University Hospital of Wales, Cardiff University, Cardiff, United Kingdom; 3 Department of Anaesthesiology and Surgical Intensive Therapy, University Medical Centre Ljubljana, Ljubljana, Slovenia; 4 Small Animal Clinic, Veterinary Faculty, University of Ljubljana, Ljubljana, Slovenia; Heidelberg University Hospital, GERMANY

## Abstract

In the obese, the evidence for the choice of the optimal emergency front-of-neck access technique is very limited and conflicting. We compared cannula and scalpel-bougie emergency front-of-neck access techniques in an anaesthetised porcine model with thick pretracheal tissue. Cannula and scalpel-bougie cricothyroidotomy techniques were performed in 11 and 12 anaesthetised pigs, respectively. Following successful tracheal access, oxygenation was commenced and continued for 5 min using Rapid-O2 device for cannula and circle breathing system for scalpel-bougie study groups. The primary outcome was a successful rescue oxygenation determined by maintenance of arterial oxygen saturation >90% 5 min after the beginning of oxygenation. Secondary outcomes included success rate of airway device placement, time to successful airway device placement, and trauma to the neck and airway. The success rate of rescue oxygenation was 18% after cannula, and 83% after scalpel-bougie technique (P = 0.003). The success rate of airway device placement was 73% with cannula and 92% with scalpel-bougie technique (P = 0.317). Median (inter-quartile-range) times to successful airway device placement were 108 (30–256) and 90 (63–188) seconds (P = 0.762) for cannula and scalpel-bougie emergency front-of-neck access, respectively. Proportion of animals with iatrogenic trauma additional to the procedure itself was 27% for cannula and 75% for scalpel-bougie technique (P = 0.039). Thus, in the porcine model of obesity, the scalpel-bougie technique was more successful in establishing and maintaining rescue oxygenation than cannula-based technique; however, it was associated with a higher risk of severe trauma.

## Introduction

The current Difficult Airway Society (DAS) guidelines recommend the scalpel-bougie cricothyroidotomy as a default technique for emergency front-of-neck access (eFONA) procedure. The guidelines, however, acknowledge that the evidence base for the choice of scalpel technique is limited and that the eFONA may indeed be performed using either a scalpel-bougie or cannula technique [1]. A systematic review of 24 studies found no advantage of any particular eFONA technique [2]. Some studies indicated that cannula technique may be the preferred option to many anaesthetists and emergency physicians as it is familiar, readily available and less invasive than the scalpel technique [3–6]. Moreover, attempting percutaneous cannula insertion (for 3 passes or 60 s) before resorting to scalpel technique in eFONA has been recommended, mindful that failed scalpel attempts may contaminate the trachea with blood preventing subsequent cannula insertion attempts [7]. Conversely, failed cannula insertion may make subsequent landmark recognition and scalpel technique more difficult if associated with subcutaneous emphysema [8]. This uncertainty of the optimal eFONA technique has led to a number of national difficult airway guidelines recommending differing eFONA techniques [9–12].

The Fourth National Audit Project (NAP4) acknowledged that serious airway morbidity is more frequent in the obese; and that eFONA techniques fail frequently in the hands of anaesthetists. The NAP4 specifically noted a high failure rate (63%) of cannula technique, mainly attributed to neck positioning, obesity, poor insertion technique, incorrect cannula ventilation method and inadequate training [13–15]. Nevertheless, when performed in a wet lab simulation using an ovine model [16], the cannula technique proved advantageous compared to the scalpel technique. Thus, given the potential impact of the thickness of the pretracheal tissue on the success of the particular eFONA technique, we set up the study using an anesthetized porcine model to compare the cannula and the scalpel-bougie techniques using the success rate of rescue oxygenation as the primary outcome measure.

## Materials and methods

### Ethical statement

The study protocol was approved by the ethical committee for animal experimentation under the administrative agency for Food Safety, Veterinary Sector and Plant Protection of the Republic of Slovenia (Licenses Numbers: U34401-20/2016/4, U34401-20/2016/9, and U34401-20/2016/13). The animals were reared in accordance with the Council of the European Union directives on minimum standards for the protection of pigs (2008/120/EC), while the overall execution of the study was in full compliance with the National Institutes of Health’s Guide for the Care and the Use of Laboratory Animals and the ARRIVE guidelines.

### Experimental animals

Twenty-three female pigs (*Sus scrofa domesticus*) crossbred from the Landrace and Large White, were obtained from a commercial farm following confirmation of their good general health status. Following the 3R principles (replacement, reduction and refinement), this study was performed in animals that were primarily included in a study of neurological and histological outcomes after subarachnoid injection of a liposomal bupivacaine suspension [17]. The animals (with mean (SD) weight of 36.9 (4.1) kg) were sheltered in straw-bedded pens and kept at room temperature of 20–29°C, relative air humidity 58–80% and natural light/dark cycle. The pigs were fed twice daily with a commercial diet preparation for growers and supplied with water from nipple waterers. General physical examination was performed to assess and certify good clinical status 12 hours prior to induction of anaesthesia.

### Study design

The animals were pre-medicated with midazolam 0.5 mg kg^-1^, butorphanol 0.5 mg kg^-1^, and ketamine 10 mg kg^-1^ mixed together and administered intramuscularly. Anaesthesia was induced and maintained with isoflurane in 100% oxygen using a facemask. Electrocardiogram (BLT M7000 VET, Guangdong Biolight Meditech, China/PRC), end-tidal carbon dioxide tension (ETCO_2_), and peripheral capillary oxygen saturation on the ear (SpO_2_) (LifeVet CP, Eickemeyer, Germany) were monitored. A 22-gauge IV cannula (BD Venflon; Becton Dickinson Infusion Therapy AB) was placed in an auricular vein and lactated Ringer’s solution infused at 10 ml kg^-1^ h^-1^. For arterial blood sampling, the femoral artery was surgically exposed and the arterial line (BD Venflon; Becton Dickinson Infusion Therapy AB) inserted and fixed.

The pigs were randomly allocated into two study groups using an online-generated sequence (GraphPad Software, CA, USA). All procedures were performed by two anaesthetists having no formal training in the two eFONA techniques within the last five years. Nevertheless, the two anaesthetists were well instructed beforehand about the performance of both eFONA techniques using reading material, Difficult Airway Society website and instructional videos [18,19]. We believe that this level of skill is likely to simulate an operator with limited emergency front-of-neck-access training. In order to replicate the ideal position required for an eFONA, the pigs were placed on their backs with sandbags under their cervico-thoracic spine to extend their necks and stabilize their body.

Rescue oxygenation was assessed by arterial blood gas analysis (S_a_O_2_ and P_a_O_2_) using RAPID Point 500 System (Siemens Healthineers Global, Erlangen, Germany). The first blood sample was taken to establish the baseline parameters ***(sample 0)***. Apnoea was then induced and maintained with boluses of propofol. The cricothyroid membrane was identified using laryngeal handshake technique as described in DAS 2015 guidelines [1]. The membrane was perceived as identified when the thyroid and cricoid cartilages were located [1]. The eFONA rescue procedure was started when SpO_2_ dropped to 80% [20]. The arterial blood sample ***(sample A)*** was taken at the start of eFONA procedure.

The eFONA procedures were performed as follows.

Cannula cricothyroidotomy technique: The 14-gauge BD Insyte^™^ cannula (BD, Franklin Lakes, NJ, USA) connected to a 5 ml syringe filled with saline was inserted in the midline through the skin, pretracheal tissue and cricothyroid membrane into the trachea, advancing while aspirating. The successful intratracheal cannula placement was confirmed by aspiration of air before and after the needle was removed. Arterial blood sample (***sample B***) was taken as the cannula was connected to the Rapid-O_2_ device (Meditech Systems Ltd, Dorset, UK) with the oxygen flow set at 15 l min^-1^ [21]. The first breath was given by closing the T piece to provide a four-second jet, followed by two-second jet (500 ml) every 15 seconds [22]. Hundred percent oxygen was delivered for 5 minutes with additional arterial blood samples taken at 3 minutes (***sample C***) and 5 minutes (***sample D***) after the beginning of oxygenation.

Scalpel-bougie cricothyroidotomy technique: A transverse skin incision using a scalpel with No. 10 blade (B. Braun, Aesculap AG, Tuttlingen, Germany) was followed by a blunt finger subcutaneous tissue dissection until the cricothyroid membrane was identified and fixed. Then, a transverse stab incision with a blade rotation of 90° with the cutting edge pointed caudally to make a triangular hole was performed. A tip of the single use bougie (Portex®, Smiths Group, MN, USA) was introduced into the trachea along the scalpel blade and advanced to the 10 cm mark, followed by insertion of a lubricated 6.0 mm ID cuffed tracheal tube (Teleflex, Rüsch, NC, USA) [23]. The successful tracheal tube placement was confirmed with capnometry_._ Arterial blood sample (***sample B***) was taken as the tracheal tube was connected to the circle breathing system (NarkoVet Anaesthetic Unit, Tuttlingen, Germany) primed with 100% oxygen to provide assisted ventilation with breathing frequency of 10 min^-1^ and tidal volume of 6–10 ml kg^-1^ using pressure-controlled ventilation set at 20 cmH_2_O. The animals were oxygenated and ventilated for 5 minutes, with arterial blood samples taken at 3 minutes (***sample C***) and 5 minutes (***sample D***) after oxygenation/ventilation started. The eFONA procedures were terminated and marked unsuccessful if the devices’ insertion times exceeded 300 seconds [22].

Primary outcome measure was the success rate of rescue oxygenation defined by the SaO_2_≥90% 5 minutes after the beginning of oxygenation/ventilation. The secondary outcomes were the success rate of airway device placement (defined by air aspiration before and after the needle removal for cannula technique and by the rise in ETCO_2_ for scalpel-bougie technique) and the time to successful airway device placement (defined by the time interval between the moment when the devices were picked up from the table to their successful airway insertion). After 5 minutes of oxygenation/ventilation, the animals were euthanized using 0.3 ml kg^-1^ intravenous T-61 euthanasia solution (1 ml containing embutramide 200 mg, mebezonium iodide 50 mg and tetracaine hydrochloride 5 mg).

After euthanasia, the necks were examined for devices’ insertion sites into the trachea, pretracheal tissue thickness (measured from the skin surface to the outer border of first tracheal cartilage); the internal tracheal diameter (measured at the level of the first tracheal ring); the incidence and the extent of injury to the skin (measured by the length of the skin wound), subcutaneous tissue (hematoma), airway (presence of the blood), injury to the major vessels; and for subcutaneous emphysema.

### Statistical analysis

Shapiro-Wilk test was used to test continuous data for normal distribution, followed by Student’s t test or Mann Whitney U test. Nominal variables were compared using Fisher’s exact test. Statistical analysis was performed with the GraphPad Prism 8 (GraphPad Software, San Diego CA, USA). The difference was considered statistically significant at *p*<0.05. The data are presented as number (%), mean (SD) or median [IQR].

## Results

**Fig 1:**
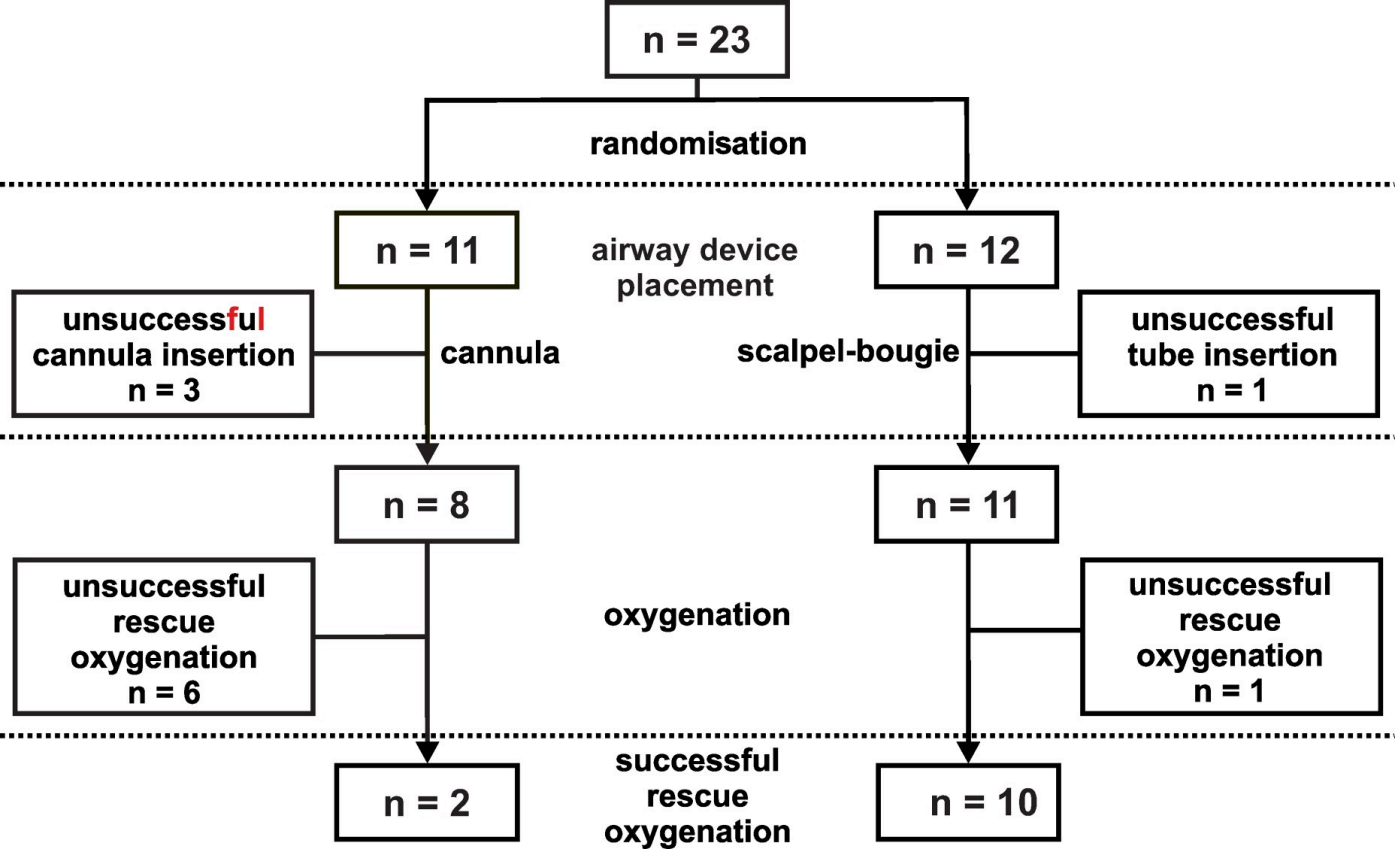
Study design. Green lines denote successful procedure while red lines denote unsuccessful procedure for emergency front-of-neck access (eFONA) using cannula or scalpel-bougie cricothyroidotomy technique.

Twenty-three animals were enrolled in the study; 11 were randomized to cannula and 12 to scalpel-bougie study groups (Fig 1). The mean (SD) pretracheal tissue thickness and internal tracheal diameter of the animals were 37 (12) mm (range 18–53 mm) and 15 (2) mm for cannula, and 34 (7) mm (range 21–48 mm) and 15 (2) mm for scalpel-bougie technique, respectively.

**Fig 2:**
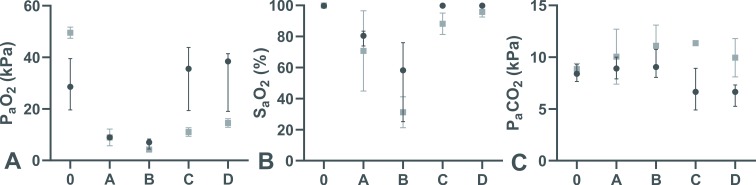
P_a_O_2_ (A), S_a_O_2_ (B) and P_a_CO_2_ (C) during successful cannula (■) and scalpel-bougie (●) emergency front-of-neck access technique attempts at different time points (0 = baseline, A = point of SpO_2_ = 80% desaturation, B = successful airway device placement/commencement of oxygenation, C = 3 minutes and D = 5 minutes after commencement of oxygenation). Data shown represent medians and interquartile ranges. *p<0.05 for cannula versus scalpel-bougie group at the same time point (Mann-Whitney U test) (n_cannula_ = 2, n_scalpel-bougie_ = 10).

The scalpel-bougie technique was found more successful and in providing rescue oxygenation and ventilation compared to cannula technique (P = 0.003) (Fig 2). In terms of the rate and the time to successful device placement, there were no significant differences observed between the two groups (Table 1).

**Table 1:** Primary and secondary outcomes of cannula and scalpel-bougie emergency front-of-neck technique study groups.

Outcomes	Cannula (n = 11)	Scalpel-bougie (n = 12)	*p* value
Success rate of rescue oxygenation	2 (18%)	10 (83%)	0.003
Success rate of airway device placement	8 (73%)	11 (92%)	0.317
Time to successful airway device placement (s)	108 [30–256]	90 [63–188]	0.762
Airway devices placed in less than 60s	3 (27%)	1 (8%)	0.317
Trauma in addition to the procedure itself	3 (27%)	9 (75%)	0.039
Presence of the tracheal wall haematoma	2 (18%)	5 (42%)	0.370
Presence of blood inside the trachea	1 (9%)	3 (21%)	0.590
Major vessel laceration	0 (0%)	1 (7%)	0.999
Death due to the procedure	0 (0%)	2 (17%)	0.478
Subcutaneous emphysema	3 (27%)	0 (0%)	0.093

Data are reported as n (%) or median [IQR]. *P* values were calculated using Fisher’s Exact test or Mann Whitney U test.

The cannula was inserted through the cricothyroid membrane in 7 (64%) animals, between cricoid and first tracheal cartilage in one (9%), while in 3 (27%) animals the entry point was not discernible. The obvious kinking of the cannula was encountered in three cases (27%). In the scalpel-bougie group, the tracheal tube insertion through the cricothyroid membrane was documented in 10 (83%) animals, and in one respective animal (8%) through the cricoid and between the cricoid and the first tracheal cartilages.

The scalpel-bougie technique was more traumatic, resulting in two life-ending injuries such as carotid artery laceration and major tracheal bleeding (Table 1, S2 and S4 Tables). Other haematomas were small and did not reduce the luminal diameter. After scalpel usage, the size of the skin wound was 27 (9) mm. After cannula insertion, on the contrary, only a small puncture wound was found in the skin and the anterior tracheal wall.

## Discussion

In an anaesthetised porcine model, a scalpel-bougie technique was more successful in establishing and maintaining rescue oxygenation as compared to the cannula technique while associated with a higher incidence of severe trauma. No significant differences were observed in the rate and the time to successful device placement between the two eFONA techniques. Since the thickness of pre-tracheal tissue in the pig model coincides with the measurements in the morbidly obese patients, the findings of this study can be extrapolated to the obese patient population [24].

In accordance with NAP4 data, our study reconfirms the low success rate of the cannula technique in the obese, primarily attributed to relative shortness of the cannula with respect to pre-tracheal tissue which increased the risk of cannula displacement and kinking despite using the 14 gauge Insyte^TM^ catheter with a good memory of initial shape [25]. The cannula was usually displaced after confirming its intratracheal placement often related to the manipulation associated with air aspiration, needle removal, and the attachment of the Rapid-O_2_ device tubing. The kink-resistant and longer cannulas might thus be of benefit in the obese neck. By contrast, a higher success rate of the scalpel-bougie technique in the obese model could be attributed to the use of the two-finger technique for blunt tissue dissection and cricothyroid membrane fixation before performing a transverse incision. In addition, the scalpel-bougie technique allowed a reliable prediction of oxygenation success from the first effective breath while obtaining a definitive eFONA with safe and secure ventilation [15]. Nevertheless, the mandatory perpendicular bougie introduction in the obese model made the insertion of coudé tip difficult which we overcame by bougie manipulation along its long axis. Using a novel cricothyroidotomy introducer invented to slide the bougie into the trachea may be of assistance in this case [26]. Our findings are therefore in contradiction to those obtained in a slim neck ovine model [16] suggesting that the pre-tracheal tissue thickness needs to be taken into consideration when deciding on the optimal eFONA technique in the patient [5].

Our study reconfirmed the impression that the scalpel technique is associated with a higher risk of severe vascular injury [25,27,28] which occurred during a stab incision through cricothyroid membrane when the scalpel blade slid laterally and lacerated the carotid artery. However, the great vessels at the level of cricothyroid membrane in the pig are positioned more medially representing a higher risk of injury as compared to those in humans [29] where no major vessels are found anterior to the cricothyroid membrane [30]. Furthermore, the cricoid cartilage injury may result in a long-term complication such as stenosis. Note that all the other tissue trauma occurred in this study (S1, S2 and S4 Tables) would most likely not have long-term clinical consequences [31,32].

Consistent with the findings in the ´wet´ sheep model of morbidly obese neck, we have not observed any differences in insertion times between the cannula and scalpel-bougie technique [25]. However, when compared to the non-obese or slim-neck animal models, longer insertion times were reported especially for cannula technique [2,13,16,25–27]. In our study, the cannula technique was successfully accomplished in less than 60 seconds in only three animals (27%) of which only one was adequately oxygenated, suggesting that attempting cannula insertion for 60 seconds before converting to scalpel technique may not be justified in the obese. Nevertheless, due to insufficient evidence, further studies of the cannula technique in obese necks using longer, kink-resistant cannulas are warranted.

Body habitus in the severely obese predisposes to greater difficulty encountered when identifying landmarks for cricothyroid puncture, particularly in a time-critical setting [33]. The increased pretracheal soft tissue depth represents an additional challenge in manually palpating the cricothyroid membrane. As a result, a cricothyroid membrane was punctured in 74% of our animals, which replicated the previous data in pig’s larynx simulating the obese neck [26]. The cricothyroid membrane identification can be improved by the use of ultrasonography. However, the factors of individual stress combined with potential technical issues connected to the ultrasound machine itself, make the use of ultrasound during emergency airway management less convenient [34]. A recent manikin-based study demonstrated the positive effect of training on the scalpel-bougie eFONA performance when the cricothyroid membrane was not palpable [35]. Consequently, there is a possibility that complications associated with the scalpel-bougie technique in our study could be avoided if regular wet lab or manikin training was implemented.

In our porcine model, the mean internal tracheal diameter was approximately 15 mm and comparable to that of the adult human trachea [36]. Dead pig laryngeal models have already been extensively used as a model of human larynx [2], given the similarity with the human anatomy [37]. To the best of our knowledge, apart from sheep, no other live anaesthetised animals have been used to systematically study emergency cricothyroidotomy procedures [16,23,25]. The sheep’s larynx is very superficial and protrudes anteriorly, making cricothyroid membrane easily palpable; sheep’s trachea is longer, more mobile with narrower intertracheal ring spaces; and infiltration of crystalloid solution subcutaneously cannot exactly mimic fat tissue [16,25,38]. Therefore, we believe that the anaesthetised pig is a better ´wet´ animal model to recreate a real-life emergency ´cannot intubate, cannot oxygenate´ situation particularly in the obese.

Our study has the following limitations: First, although we waited until the animals desaturated to SpO_2_ of 80% before commencing the eFONA procedure, it was very likely that the controlled lab-environment may not have adequately reflected the variables of clinical emergency circumstances. Second, we slightly modified the oxygen jetting technique using RapidO2 to deliver oxygen every 15 seconds irrespective of SpO_2_ reading to standardize delivered oxygen volume. Third, performing the same procedure multiple times might have had a learning effect; however, each anaesthetist performed both procedures the same number of times in a random order, reducing potential bias. Fourth, due to small sample size, the results should be interpreted and translated with caution. Further studies with larger sample size are therefore warranted. Fifth, in pigs, the sternohyoid muscle overlays the larynx which could affect the handling and bleeding of the pretracheal tissue. Finally, similar to a recently published study we did not paralyze our animal models [16]. Despite that, the animals were deeply anaesthetised with propofol with no spontaneous breathing efforts observed in any animal.

In an obese animal model, the scalpel-bougie technique was more successful in providing rescue oxygenation than cannula technique. However, caution is advised with scalpel-bougie technique due to the higher risk of severe trauma. The results suggest that the pre-tracheal tissue thickness needs to be taken into consideration when deciding on the eFONA technique.

## Supporting information

S1 Table Time to device placement and results of arterial gas analysis after scalpel-bougi emergency front of neck access at different time points (0 = baseline, A = point of SpO2 = 80% desaturation, B = successful airway device placement/commencement of oxygenation, C = 3 minutes and D = 5 minutes after commencement of oxygenation).
(DOCX)

S2 Table Skin wound length, pretracheal tissue thickness, internal tracheal diameter, entry point of tracheal device and trauma after scalpel-bougie emergency front of neck access. CM Cricothyroid membrane
(DOCX)

S3 Table Time to device placement and results of arterial blood gas analysis after cannula emergency front of neck access at different time points (0 = baseline, A = point of SpO2 = 80% desaturation, B = successful airway device placement/commencement of oxygenation, C = 3 minutes and D = 5 minutes after commencement of oxygenation). (DOCX)

S4 Table Skin wound length, pretracheal tissue thickness, internal tracheal diameter, entry point of tracheal device and trauma after cannula emergency front of neck access. CM Cricothyroid membrane, ND Not discernible
(DOCX)
