# Supplementary material for: Rescue oxygenation success by cannula or scalpel-bougie emergency front-of-neck access in an anaesthetised porcine model
Source: PLoS One. 2020 May 4;15(5):e0232510. doi: 10.1371/journal.pone.0232510 (PMC7197851; doi:10.1371/journal.pone.0232510)
Supplement: S1 Table — Time to device placement and results of arterial gas analysis after scalpel-bougi emergency front of neck access at different time points (0 = baseline, A = point of SpO2 = 80% desaturation, B = successful airway device placement/commencement of oxygenation, C = 3 minutes and D = 5 minutes after commencement of oxygenation). (DOCX) [file pone.0232510.s001.docx]

|  | | | | 0 | | | A | | | B | | | C | | | D | | |
| --- | --- | --- | --- | --- | --- | --- | --- | --- | --- | --- | --- | --- | --- | --- | --- | --- | --- | --- |
| Animal No. | eFONA technique | Provider | Time  (s) | p_a_O_2_  (kPa) | p_a_CO_2_  (kPa) | S_a_O_2_  (%) | p_a_O_2_  (kPa) | p_a_CO_2_  (kPa) | S_a_O_2_  (%) | p_a_O_2_  (kPa) | p_a_CO_2_  (kPa) | S_a_O_2_  (%) | p_a_O_2_  (kPa) | p_a_CO_2_  (kPa) | S_a_O_2_  (%) | p_a_O_2_  (kPa) | p_a_CO_2_  (kPa) | S_a_O_2_  (%) |
| 1 | scalpel | MD | 250 | 29.4 | 8.4 | 99.8 | 8.4 | 7.6 | 80.1 | 4.2 | 7.9 | 26.8 | 5.7 | 10.5 | 39.8 | 12.3 | 6.9 | 93.9 |
| 2 | scalpel | TSP | 102 | 12.7 | 8.0 | 92.8 | 5.6 | 9.9 | 84.4 | 8.2 | 8.9 | 71.7 | 20.7 | 6.3 | 99.7 | 23.2 | 5.3 | 99.7 |
| 3 | scalpel | TSP | 188 | 27.5 | 11.0 | 99.7 | 9.2 | 8.7 | 80 | 3.9 | 11.2 | 20.1 | 41.6 | 7.0 | 99.8 | 40.9 | 6.5 | 99.8 |
| 4 | scalpel | MD | 63 | 27.7 | 8.4 | 99.7 | 8.8 | 8.4 | 82.2 | 8.3 | 7.6 | 76.5 | 33.9 | 4.5 | 99.8 | 43.0 | 5.1 | 99.8 |
| 5 | scalpel | TSP | 90 | 12.4 | 6.2 | 99.9 | 9.2 | 7.4 | 83.1 | 4.5 | 10.7 | 44.7 | 15.3 | 8.8 | 99.9 | 11.8 | 7.7 | 99.9 |
| 6 | scalpel | MD | 110 | 20.6 | 8.9 | 99.7 | 7.5 | 8.9 | 72.2 | 5.8 | 8.5 | 52.4 | 5.7 | 6.6 | 38.1 | 5.7 | 6.3 | 29.9 |
| 7 | scalpel | TSP | 52 | 41.9 | 8.7 | 92.2 | 10.9 | 10.6 | 76.8 | 11.6 | 9.2 | 20.1 | 37.2 | 8.5 | 96 | 39.9 | 7.2 | 90.1 |
| 8 | scalpel | MD | 299 | 34.1 | 6.9 | 99.9 | 8.1 | 8.0 | 65.3 | 8.4 | 8.1 | 90.8 | 50.5 | 4.9 | 99.8 | 36.9 | 4.2 | 99.9 |
| 9 | scalpel | TSP | 82 | 46.9 | 8.7 | 99.9 | 12.7 | 10.3 | 92.4 | 5.9 | 10.1 | 75.9 | 21.3 | 9.3 | 99.8 | 21.3 | 9.7 | 99.9 |
| 10 | scalpel | MD | 81 | 21.9 | 7.9 | 99.9 | 7.6 | 9.1 | 59.8 | 7.9 | 8.5 | 43.7 | 52.4 | 4.9 | 99.8 | 45.4 | 6.1 | 99.6 |
| 11 | scalpel | MD | Death |  |  |  |  |  |  |  |  |  |  |  |  |  |  |  |
| 12 | scalpel | TSP | 61 | 38.7 | 11.0 | 99.7 | 9.0 | 9.3 | 80.9 | 6.2 | 10.9 | 74.5 | 39.3 | 4.9 | 99.9 | 40.5 | 6.8 | 99.9 |

**S1 Table** Time to device placement and results of arterial gas analysis after scalpel-bougi emergency front of neck access at different time points (0=baseline, A=point of SpO2=80% desaturation, B=successful airway device placement/commencement of oxygenation, C=3 minutes and D=5 minutes after commencement of oxygenation).
