# Supplementary material for: Rescue oxygenation success by cannula or scalpel-bougie emergency front-of-neck access in an anaesthetised porcine model
Source: PLoS One. 2020 May 4;15(5):e0232510. doi: 10.1371/journal.pone.0232510 (PMC7197851; doi:10.1371/journal.pone.0232510)
Supplement: S2 Table — CM Cricothyroid membrane (DOCX) [file pone.0232510.s002.docx]

**S2 Table** Skin wound length, pretracheal tissue thickness, internal tracheal diameter, entry point of tracheal device and trauma after scalpel-bougie emergency front of neck access. CM Cricothyroid membrane

| Animal No. | eFONA technique | Provider | Skin wound (mm) | Pretracheal tissue thickness (mm) | Tracheal diameter  (mm) | Entry point | Trauma |
| --- | --- | --- | --- | --- | --- | --- | --- |
| 1 | scalpel | MD | 33 | 33 | 14 | CM | Dorsal tracheal wall haematoma |
| 2 | scalpel | TSP | 25 | 38 | 16 | CM | Dorsal tracheal wall haematoma |
| 3 | scalpel | TSP | 48 | 21 | 16 | CM | Minimal amount of blood inside trachea |
| 4 | scalpel | MD | 30 | 25 | 12 | Before first tracheal ring | Dorsal tracheal wall haematoma |
| 5 | scalpel | TSP | 40 | 30 | 17 | Cricoid cartilage |  |
| 6 | scalpel | MD | 15 | 28 | 14 | CM | Sever tracheal bleeding, voluminous amounts of blood inside trachea  Death due to procedure |
| 7 | scalpel | TSP | 18 | 34 | 14 | CM |  |
| 8 | scalpel | MD | 25 | 38 | 16 | CM | Dorsal tracheal wall haematoma |
| 9 | scalpel | TSP | 18 | 38 | 18 | CM | Dorsal tracheal wall haematoma |
| 10 | scalpel | MD | 17 | 48 | 14 | CM |  |
| 11 | scalpel | MD | 25 | 43 | 16 | CM | Carotid laceration  Death due to procedure |
| 12 | scalpel | TSP | 24 | 33 | 17 | CM | Minimal amount of blood inside trachea |
