# Supplementary material for: Rescue oxygenation success by cannula or scalpel-bougie emergency front-of-neck access in an anaesthetised porcine model
Source: PLoS One. 2020 May 4;15(5):e0232510. doi: 10.1371/journal.pone.0232510 (PMC7197851; doi:10.1371/journal.pone.0232510)
Supplement: S3 Table — (DOCX) [file pone.0232510.s003.docx]

**S3 Table** Time to device placement and results of arterial blood gas analysis after cannula emergency front of neck access at different time points (0=baseline, A=point of SpO2=80% desaturation, B=successful airway device placement/commencement of oxygenation, C=3 minutes and D=5 minutes after commencement of oxygenation).

|  | | | | 0 | | | A | | | B | | | C | | | D | | |
| --- | --- | --- | --- | --- | --- | --- | --- | --- | --- | --- | --- | --- | --- | --- | --- | --- | --- | --- |
| Animal No. | eFONA technique | Provider | Time  (s) | p_a_O_2_  (kPa) | p_a_CO_2_  (kPa) | S_a_O_2_  (%) | p_a_O_2_  (kPa) | p_a_CO_2_  (kPa) | S_a_O_2_  (%) | p_a_O_2_  (kPa) | p_a_CO_2_  (kPa) | S_a_O_2_  (%) | p_a_O_2_  (kPa) | p_a_CO_2_  (kPa) | S_a_O_2_  (%) | p_a_O_2_  (kPa) | p_a_CO_2_  (kPa) | S_a_O_2_  (%) |
| 13 | cannula | MD | 46 | 41.9 | 9.9 | 99.8 | 12.4 | 9.9 | 92.4 | 9.4 | 9.7 | 81.9 | 8.9 | 10.9 | 78.2 | 8.3 | 10.6 | 74.3 |
| 14 | cannula | MD | 76 | 34.6 | 9.3 | 99.8 | 8.4 | 9.07 | 76.1 | 3.4 | 12.1 | 13.4 | 6.2 | 16.7 | 45.7 | 3.7 | 17.3 | 15.1 |
| 15 | cannula | TSP | 18 | 29.3 | 8.3 | 99.8 | 8.5 | 12.4 | 74.5 | 4.5 | 14.0 | 27.1 | 6.5 | 17.6 | 46.2 | 5.2 | 16.0 | 30.6 |
| 16 | cannula | MD | 300 | 27.7 | 7.90 | 99.7 | 8.9 | 10.3 | 67.2 | 4.2 | 17.3 | 16.5 | 6.3 | 22.1 | 32.4 | 5.2 | 19.0 | 21.6 |
| 17 | cannula | TSP | 295 | 38.9 | 8.6 | 99.9 | 8.1 | 10.4 | 69.6 | 4.5 | 11.8 | 18.4 | 3.4 | 12.4 | 22.4 | 6.7 | 13.6 | 40.3 |
| 18 | cannula | MD | unsuccessful |  |  |  |  |  |  |  |  |  |  |  |  |  |  |  |
| 19 | cannula | TSP | 140 | 51.7 | 8.3 | 99.9 | 12.2 | 7.4 | 96.6 | 4.5 | 9.1 | 41.2 | 12.7 | 11.1 | 95.1 | 16.2 | 8.1 | 99.2 |
| 20 | cannula | MD | 25 | 47.4 | 9.4 | 99.9 | 5.7 | 12.7 | 44.9 | 4.0 | 13.1 | 21.2 | 9.4 | 11.6 | 81.3 | 12.9 | 11.8 | 92.5 |
| 21 | cannula | TSP | 140 | 47.7 | 8.2 | 99.8 | 9.4 | 9 | 83 | 11.7 | 9.7 | 32.4 | 5.1 | 11.2 | 40 | 2.6 | 11.6 | 11.7 |
| 22 | cannula | TSP | unsuccessful |  |  |  |  |  |  |  |  |  |  |  |  |  |  |  |
| 23 | cannula | MD | unsuccessful |  |  |  |  |  |  |  |  |  |  |  |  |  |  |  |
