# Supplementary material for: Rescue oxygenation success by cannula or scalpel-bougie emergency front-of-neck access in an anaesthetised porcine model
Source: PLoS One. 2020 May 4;15(5):e0232510. doi: 10.1371/journal.pone.0232510 (PMC7197851; doi:10.1371/journal.pone.0232510)
Supplement: S4 Table — CM Cricothyroid membrane, ND Not discernible (DOCX) [file pone.0232510.s004.docx]

| Animal No. | eFONA technique | Provider | Skin wound (mm) | Pretracheal tissue thickness (mm) | Tracheal diameter  (mm) | Entry point | Trauma |
| --- | --- | --- | --- | --- | --- | --- | --- |
| 13 | cannula | MD | puncture | 50 | 16 | CM |  |
| 14 | cannula | MD | puncture | 47 | 18 | Before first tracheal ring | Ventral tracheal wall haematoma |
| 15 | cannula | TSP | puncture | 43 | 17 | CM |  |
| 16 | cannula | MD | puncture | 26 | 14 | ND | Subcutaneous emphysema |
| 17 | cannula | TSP | puncture | 45 | 15 | CM |  |
| 18 | cannula | MD | puncture | 25 | 13 | ND | Ventral tracheal wall haematoma |
| 19 | cannula | TSP | puncture | 18 | 11 | CM |  |
| 20 | cannula | MD | puncture | 53 | 18 | CM |  |
| 21 | cannula | TSP | puncture | 35 | 13 | ND | Subcutaneous emphysema |
| 22 | cannula | TSP | puncture | 26 | 14 | CM | Minimal amount of blood inside trachea |
| 23 | cannula | MD | puncture | 34 | 14 | CM | Subcutaneous emphysema |

**S4 Table** Skin wound length, pretracheal tissue thickness, internal tracheal diameter, entry point of tracheal device and trauma after cannula emergency front of neck access. CM Cricothyroid membrane, ND Not discernible
